# Open-loop narrowband magnetic particle imaging based on mixed-frequency harmonic magnetization response

**DOI:** 10.3389/fmedt.2024.1464780

**Published:** 2024-10-23

**Authors:** Hongli Yu, Ping Huang, Xiting Peng, Zheyan Wang, Zhichuan Qiu, Kewen Li, Tianshu Li, Zhiyao Liu, Hao Cui, Shi Bai

**Affiliations:** School of Information Science and Engineering, Shenyang University of Technology, Shenyang, China

**Keywords:** MPI, SNR, superparamagnetic nanoparticles, mixed-frequency harmonic magnetization response, narrowband

## Abstract

**Introduction:**

Magnetic particle imaging (MPI), a radiation-free, dynamic, and targeted imaging technique, has gained significant traction in both research and clinical settings worldwide. Signal-to-noise ratio (SNR) is a crucial factor influencing MPI image quality and detection sensitivity, and it is affected by ambient noise, system thermal noise, and the magnetization response of superparamagnetic nanoparticles. Therefore to address the high amplitude system and inherent thermal noise present in conventional MPI systems is essential to improve detection sensitivity and imaging resolution.

**Method:**

This study introduces a novel open-loop, narrow-band MPI signal acquisition system based on mixed-frequency harmonic magnetization response. Allowing superparamagnetic nanoparticles to be excited by low frequency, high amplitude magnetic fields and high frequency, low amplitude magnetic fields, the excitation coil generates a mixed excitation magnetic field at a mixed frequency of 8.664 kHz (*f*_*H*_ + 2*f*_*L*_), and the tracer of superparamagnetic nanoparticles can generate a locatable superparamagnetic magnetization signal with rich harmonic components in the mixed excitation magnetic field and positioning magnetic field. The third harmonic signal is detected by a Gradiometer coil with high signal-to-noise ratio, and the voltage cloud image is formed.

**Result:**

The experimental results show that the external noise caused by the excitation coil can be effectively reduced from 12 to about 1.5 μV in the imaging area of 30 mm × 30 mm, which improves the stability of the detection signal of the Gradiometer coil, realizes the detection of high SNR, and makes the detection sensitivity reach 10 μg Fe. By mixing excitation, the total intensity of the excitation field is reduced, resulting in a slight improvement of the resolution under the same gradient field, and the spatial resolution of the image reconstruction is increased from 2 mm under the single frequency excitation (20.7 kHz) in the previous experiment to 1.5 mm under the mixed excitation (8.664 kHz).

**Conclusions:**

These experimental results highlight the effectiveness of the proposed open-loop narrowband MPI technique in improving signal detection sensitivity, achieving high signal-to-noise ratio detection and improving the quality of reconstructed images by changing the excitation magnetic field frequency of the excitation coil, providing novel design ideas and technical pathways for future MPI systems.

## 1 Introduction

Magnetic particle imaging (MPI), a non-invasive technique exploiting the nonlinear magnetic properties of superparamagnetic nanoparticles, was introduced in Nature in 2005 by Gleich et al. (1). It offers radiation-free, dynamic, targeted imaging of deep biological tissues (2–4). With demonstrated advantages in spatial and temporal resolution, as well as biocompatibility, MPI has garnered substantial preclinical interest across applications such as tumor imaging, cardiovascular monitoring, and cell tracking (5, 6). This has led to widespread adoption within the research and clinical communities.

While significant advancements have been made in MPI hardware, signal processing, and multimodal imaging (7–9), translating the technology to clinical applications, particularly at the human scale, remains challenging due to aperture size limitations (6). Current MPI systems, exemplified by those from Berkeley and Philips (10), typically possess apertures of 3–5 cm, suitable only for small animal models. To address this, Graeser et al. (11) pioneered human-scale MPI brain imaging, demonstrating rapid acquisition of high-quality cerebral perfusion data for monitoring post-operative stroke and related vascular conditions. Additionally, multimodal imaging combining MPI with magnetic resonance imaging (MRI) has shown promise, leveraging MPI's strengths in vascular and inflammation imaging with MRI's high-resolution soft tissue capabilities (12–16).

This study focuses on enhancing signal detection and data acquisition within MPI. A novel open-loop narrowband MPI technique is proposed, leveraging a mixed-frequency harmonic magnetization response to improve signal-to-noise ratio (SNR), a critical performance metric in MPI (17). Conventional MPI systems often employ high-amplitude, high-frequency AC signals to amplify the magnetization response of superparamagnetic nanoparticles, relying on broadband signal detection for SNR enhancement (18). However, this approach introduces significant system noise and thermal noise due to the intense excitation (19).

To address these limitations, we introduce a mixed-frequency excitation scheme that combines high-amplitude, low-frequency and low-amplitude, high-frequency AC magnetic fields to induce a robust nonlinear magnetization response in superparamagnetic nanoparticles even under low-power conditions (20, 21). Concurrently, narrowband signal detection effectively suppresses fundamental wave noise and ambient interference, leading to a substantial SNR improvement.

This paper presents a novel Magnetic Particle Imaging (MPI) scanner featuring a unique bilaterally arranged electromagnetic scanning magnetic field, creating an open scanning structure. This design is well-suited for large-scale imaging applications, offering enhanced flexibility and adaptability. The system employs a narrow-band detection method to effectively extract the characteristic third-harmonic magnetization response signal generated by the tracer under mixed-frequency excitation conditions. The detected signal is transmitted to a personal computer (PC) for the creation of a voltage cloud map, providing raw data for subsequent image reconstruction. To mitigate external noise generated by the excitation coil, a mixed-frequency excitation method (*f_H_* + 2*f_L_*) is employed, involving variation of the excitation magnetic field frequency. Furthermore, an MPI imaging system incorporating a superparamagnetic tracer subjected to special magnetic screening has been developed. This system comprises a data acquisition system, a spatial localization system, and an image reconstruction system. The study not only introduces a new open MPI imaging technology but also experimentally validates its imaging performance, demonstrating significant potential to expand the application of MPI technology in biomedical imaging.

## 2 Mixed frequency narrowband measurement

### 2.1 Narrowband measurements

Conventional broadband MPI systems typically employ a 25.5 kHz excitation frequency with a bandwidth exceeding 500 kHz (4, 7, 11). This necessitates the use of untuned receiver coils, compromising optimal preamplifier-receiver coil matching. In contrast, the proposed narrowband MPI technique focuses on detecting and processing a specific frequency band, enhancing imaging sensitivity and selectivity while mitigating signal interference from extraneous magnetic materials (22). By precisely aligning the driving field and receiver coil frequency bands, the signal-to-noise ratio is significantly improved, leading to enhanced image quality (23, 24).

### 2.2 Mixed frequency harmonic signal response and acquisition

In narrowband MPI, the signal response of magnetic nanoparticles can be modeled using two primary approaches (12, 25, 26). The first employs a computationally intensive model encompassing both linear and saturation magnetization regions, characterized by a function *F*(*x*) (strength of the particle signal response). Additional approach utilizes functions *f*(*x*) (induced voltage signal) and *G*(*x*) (a linear fit to the gradient magnetic field) to describe the particle's signal response under field-free conditions and the influence of the saturated DC magnetic field, respectively. This latter method can directly incorporate experimental data through *G*(*x*), establishing a practical link between hardware, material properties, and signal response. It simplifies computations while accurately representing particle behavior under mixed-frequency harmonic excitation. Consequently, this study adopts the second approach to model particle signal response.

**Figure 1:**
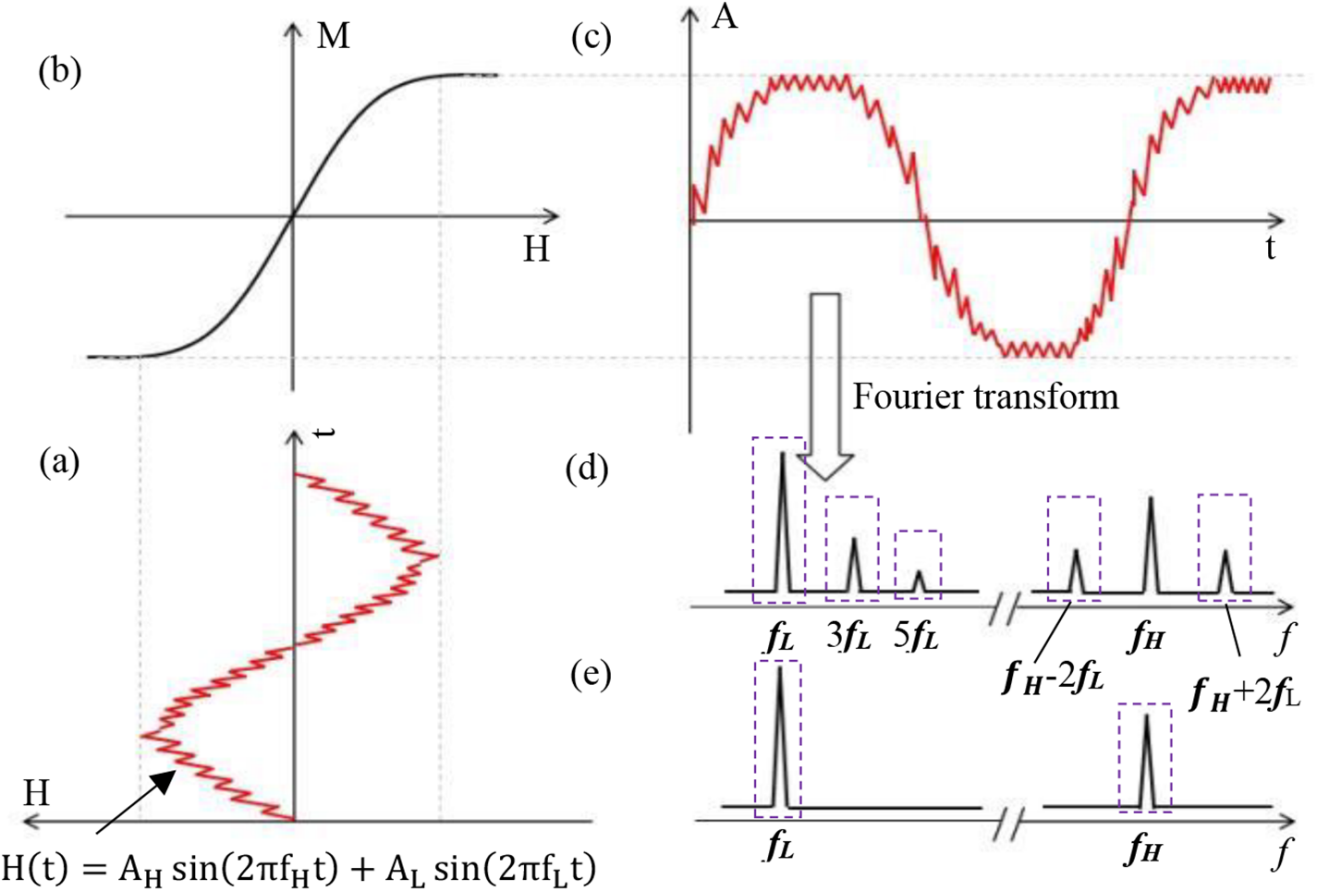
Magnetization response process based on a double-excitation magnetic field. **(a)** Time trajectory of a magnetic particle exposed to a magnetic field consisting of two frequency components (***f_H_*** and ***f_L_***). **(b)** Magnetization signal response curve of a magnetic nanoparticle. **(c)** Time-varying magnetization response of a magnetic nanoparticle. **(d)** Higher harmonics and frequency mixing components. **(e)** Excitation spectrum showing two different frequency lines.

Under mixed-frequency excitation, superparamagnetic nanoparticles (SPIONs) experience simultaneous stimulation from two AC magnetic fields of differing frequencies and amplitudes. Figure 1 illustrates the resulting magnetization response of SPIONs under these conditions.

The magnetization intensity profile of SPIONs in solution can be approximated by the Langevin function (1), as depicted in Figure 1.

(1) $$\frac{M}{M_{S}}=L\left(\frac{m_{0}\mu_{0}H}{k_{B}T}\right)$$

The preceding Equation 1 employs ***m*_0_** to represent the magnetic moment of a single particle, ***μ*_0_** for vacuum permeability, ***K_B_*** for the Boltzmann constant, ***T*** for absolute temperature, and ***M_S_*** for the particle's saturation magnetization.

The Langevin function, denoted as L(ξ) (1, 2), is expressed by Equation 2 as follows:

$$L(\xi )=\coth(\xi )-\left(\frac{1}{\xi }\right)$$

(2) $$\xi =\frac{m_{0}\mu_{0}H}{k_{B}T}$$

Where ***ξ*** represents the ratio of maximum external field energy to thermal energy.

Assuming a time-varying magnetic field comprising high-frequency (***f_H_***) and low-frequency (***f_L_***) components (17), the dual-frequency excitation trajectory is depicted in Figure 1a.

(3) $$H(t)=A_{H}\sin(2\pi f_{H}t)+A_{L}\sin(2\pi f_{L}t)$$

In the Equation 3, ***A_H_*** and ***A_L_*** represent the high and low-frequency amplitudes, respectively. Neglecting linear response, DC field effects, and relaxation phenomena, and applying the Langevin function followed by Taylor expansion to the time-domain mixed-frequency field ***H*(*t*)**, the magnetization intensity ***M*** in the frequency domain can be derived (17). The magnetization response signal at frequency ***f_H_*** + **2*f_L_*** is expressed by Equation 4 as follows:

(4) $$\begin{array}{rl} \frac{M}{M_{S}} & =L\left(\frac{m_{0}\mu_{0}H}{k_{B}T}\right)=\frac{m_{0}\mu_{0}}{3k_{B}T}H-\frac{1}{45}{\left(\frac{m_{0}\mu_{0}}{k_{B}T}\right)}^{3}H^{3}+\cdots \\ & =\cdots +\frac{1}{60}{\left(\frac{m_{0}\mu_{0}}{k_{B}T}\right)}^{3}A_{H}A_{L}^{2}\\ & \;\times \{\cos[2\pi (\;f_{H}+2f_{L})t]+\cos[2\pi (\;f_{H}-2f_{L})t]\}+\cdots \end{array}$$

According to Faraday's law of induction, in the absence of a DC gradient magnetic field, the induced voltage signal $u^{P}$ exhibits a linear relationship with the magnetization strength, ***M***, of the MNPs at the frequency (***f_H_* -** 2***f_L_***), it can be expressed by Equation 5.

$$u^{P}=\frac{dM}{dt}=\frac{\pi }{30}{\left(\frac{m_{0}\mu_{0}}{k_{B}T}\right)}^{3}A_{H}A_{L}^{2}\times \left\{(\;f_{H}-2f_{L})\cos[2\pi (\;f_{H}-2f_{L})t]+\frac{3}{2}\pi \right\}$$

(5) $$f(x)=\frac{\pi }{30}{\left(\frac{m_{0}\mu_{0}}{k_{B}T}\right)}^{3}A_{H}A_{L}^{2}\times \left\{(\;f_{H}-2f_{L})\cos[2\pi (\;f_{H}-2f_{L})t]+\frac{3}{2}\pi \right\}$$

To further investigate the relationship between the mixed-frequency harmonic signal and the gradient magnetic field under a DC gradient field, the real function ***G*(*x*)** (12, 25, 26) is defined Equation 6 as follows:

(6) $$u^{P}=G(x)\cdot f(x)$$

The real function *G*(*x*) is defined in Section 2.3.

### 2.3 Measurement of mixed frequency harmonic signals

In narrowband MPI, harmonic signals are generated from magnetic nanoparticles (MNPs) using an AC excitation field, while a DC gradient field establishes field-free points. To optimize system design, the relationship between the mixed-frequency harmonic signal and the DC field (direction and amplitude) must be characterized.

To effectively excite magnetic nanoparticles while minimizing interference from the background power frequency (0.05 kHz), the low-frequency magnetic field excitation frequency was set to 0.102 kHz, slightly exceeding the power frequency. To determine the optimal high-frequency excitation frequency, a controlled-variable approach was employed. Systematic observation of the magnetic nanoparticles’ response characteristics was conducted by adjusting the high-frequency excitation signal frequency. The optimal high-frequency magnetic field frequency, chosen based on system performance, safety regulations, and experimental accuracy, was identified as the frequency that elicited the strongest signal response.

Five independent experiments were designed to establish the optimal high-frequency excitation frequency (*f_H_*) and its current intensity (*A_H_*). Each experiment utilized 5 μl of Resovist sample at a concentration of 27.875 mg/ml. To ensure consistent experimental conditions, the low-frequency excitation parameters were held constant at a current (*A_L_*) of 15 amperes and a frequency (*f_L_*) of 0.102 kHz across all experimental groups. This standardized setting minimized the influence of low-frequency variables on signal measurement, thereby facilitating a more precise evaluation of the high-frequency excitation parameters’ impact on the signal.

**Figure 2:**
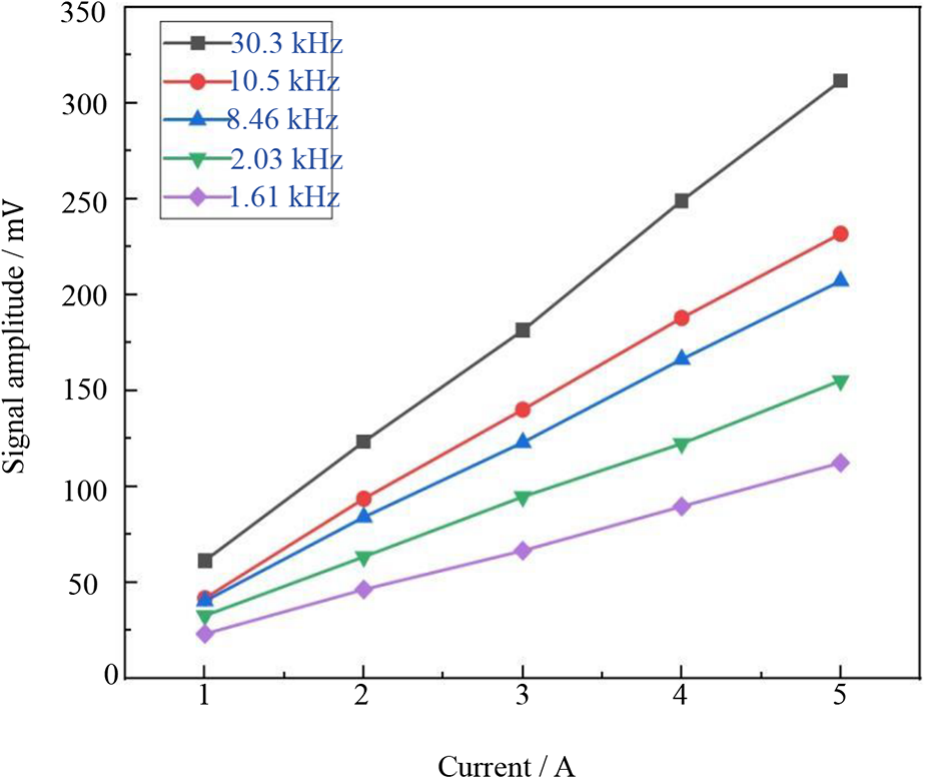
Signal variation at different currents/frequencies.

The high-frequency excitation frequencies (*f_H_*) for the five experiments were set at 1.61, 2.03, 8.46, 10.5, and 30.3 kHz, respectively. Within each group, the high-frequency excitation signal current was progressively increased from 1 to 5 A while maintaining a constant frequency. The amplitude data series of the third harmonic signal generated by mixing were meticulously recorded in Figure 2, providing a visual representation of the dynamic changes in the mixed third harmonic signal under varying high-frequency excitation parameters. These data serve as a robust foundation for subsequent in-depth analysis of Resovist's signal response characteristics under different high-frequency excitation conditions, ultimately enabling the precise determination of the optimal excitation frequency and current amplitude for the experiment.

Figure 2 demonstrates a significant enhancement in the detection signal amplitude as the excitation current increases, while maintaining a constant high-frequency excitation frequency. However, while increasing the current improves signal response, it also leads to greater heating of the experimental coil and consequently, higher instrument thermal noise levels. To balance thermal stability and operational safety, a safe working threshold of 20 A was established. A high-frequency excitation current of 3 A was selected, ensuring sufficient magnetization response signal strength and a favorable signal-to-noise ratio.

The mixing experiment aims to optimize signal response, minimize external noise interference, and enhance detection sensitivity. Given that the excitation frequency consists of two components—a high frequency (*f_H_*) and a low frequency (*f_L_*)—selecting an appropriate frequency combination is essential for extracting a high-quality detection signal. To further improve the clarity of the mixed detection signal, It is necessary to make the combined frequency (*f_H_* + 2*f_L_*) signal response larger relative to the high frequency (*f_H_*). Through optimization of this combined frequency and repeated comparative experiments, a high frequency (*f_H_*) of 8.460 kHz was determined, successfully maximizing the amplitude and sensitivity of the detection signal.

**Figure 3:**
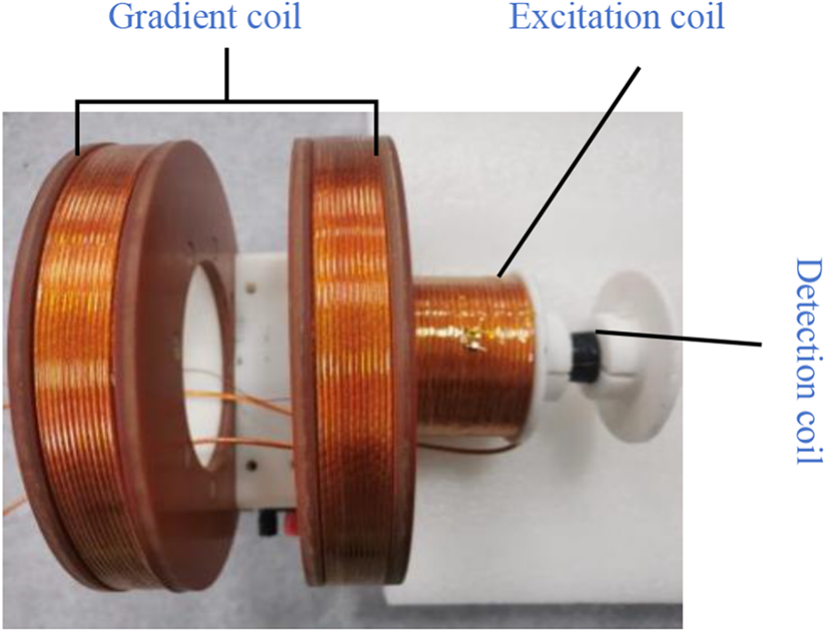
Mixed frequency harmonic measurement system.

The experimental setup depicted in Figure 3 was utilized to investigate the relationship between the mixed harmonic signal and the DC field, including its direction and strength. The device consists of a cylindrical electromagnetic coil. During the experiment, two excitation coils were simultaneously subjected to a mixed excitation signal: a low-frequency signal (0.102 kHz, 15 A) and a high-frequency signal (8.460 kHz, 3 A). Under the combined influence of these signals, the excitation coil generated a sinusoidal excitation magnetic field of approximately 3 mT, which interacted with the MNP sample.

Upon exposure to the excitation field, the magnetic nanoparticles generate a mixed-frequency harmonic signal at *f_H_* + 2*f_L_* (where *f_H_* and *f_L_* represent the high and low-frequency components, respectively). This signal is captured and recorded by a differential detection coil. A pair of Helmholtz coils, each with a diameter of 240 mm and separated by 120 mm, was employed to generate a DC gradient field. The actual gradient coils were fabricated using 3D printing of two 100 mm diameter thermoset acrylic resin formers. These coils were wound with 218 turns of 0.1 mm * 300 excitation wire, resulting in an average DC resistance of 0.83 Ω for both gradient wires. The adjustable gradient field ranged from −40 to 40 mT.

The AC excitation coil was wound with 120 turns of 0.1 mm * 350 strands of excitation wire on a former (the diameter equals to 40 mm). The LCR tester measured an inductance of 1.412 mH, a resistance of 0.4 Ω, and a calculated series resonance capacitance of 41.866 nF.

The differential receiving coil was constructed using copper enameled wire, consisting of two coils wound in opposite directions and connected in series. The detection coil former had a diameter of 20 mm, and the copper enameled wire had a diameter of 0.1 mm. With 500 turns, the receiving coil exhibited an inductance of 0.98 mH and a resistance of 1.3 Ω. To enhance signal voltage sensitivity, a resonant capacitor C was connected in parallel with the detection coil. This parallel LC resonant circuit amplified the voltage signal at the specific frequency of 8.664 kHz, enabling the receiving coil to detect the magnetization response signal of the magnetic nanoparticles more efficiently and rapidly, thereby optimizing signal output.

**Figure 4:**
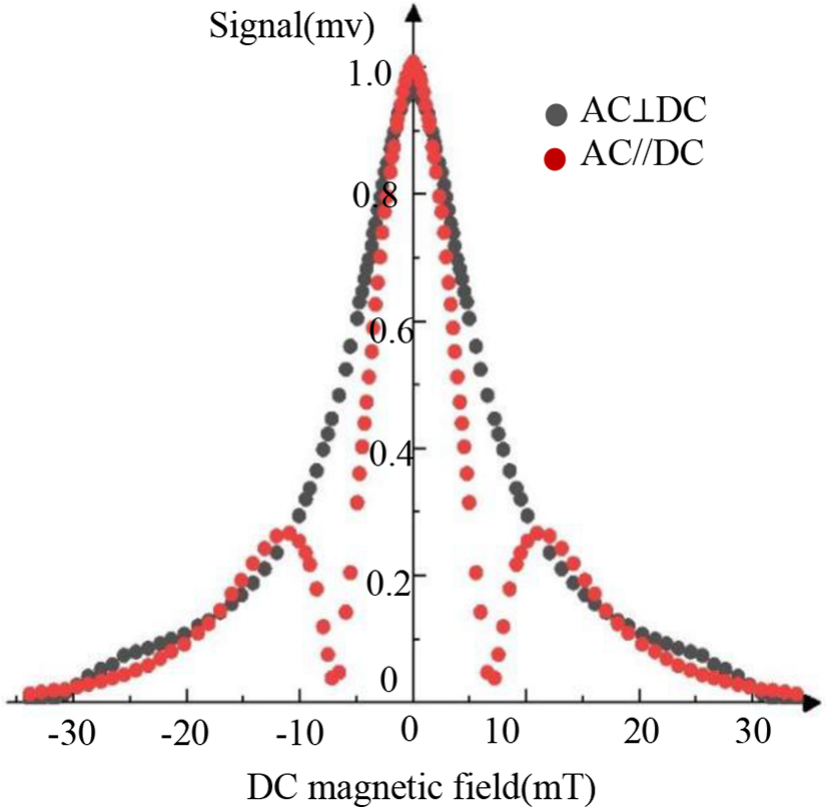
Dependence of a mixed harmonic signal on the DC field.

In the experiment, the dependence of the harmonic signal on the DC field was measured under the conditions of ***H_ac_***//***H_dc_*** and ***H_ac_***⊥***H_dc_***, respectively. Figure 4 shows the results of the mixed 3rd harmonic signal under two conditions, where the mixing excitation frequency is ***f_H_*** + 2***f_L_***. In the figure, the vertical axis represents the mixed harmonic signal normalized by the value at ***B_dc_*** = 0, and the abscissa represents the field strength of the DC magnetic field ***B_dc_***.

As illustrated in Figure 4, the third harmonic signal exhibits distinct ***B_dc_***-dependent behavior for the parallel (***H_ac_***//***H_dc_***) and perpendicular (***H_ac_***⊥***H_dc_***) configurations. In the parallel case, a double-peaked signal is observed at ***B_dc_*** = 0 mT and ***B_dc_*** = ±11 mT, followed by a gradual decay. This behavior aligns with Langevin function predictions. Conversely, the perpendicular configuration yields a single peak at ***B_dc_*** = 0 mT with a monotonic decrease in signal amplitude as ***B_dc_*** increases, contrasting with the parallel case. Given the potential image artifacts associated with the double-peaked signal in the parallel configuration, the perpendicular arrangement is adopted for the narrowband MPI system investigated in this study.

To determine the real function ***G*(*x*)**, the harmonic signals under ***H_ac_***⊥***H_dc_*** conditions were subjected to normalization and subsequent processing, as illustrated in Figure 4. A Lorentzian function was iteratively fitted to the data using the Levenberg-Marquardt algorithm. As Equation 7 follow:

(7) $$G(x)=\frac{2A}{\pi }\cdot \frac{\omega }{4x^{2}+\omega^{2}}$$

In the equation, ***ω*** represents the DC magnetic field range corresponding to the half-maximum signal amplitude, ***A*** is a system-dependent constant, and ***x*** denotes the DC magnetic field magnitude. The mixed-frequency harmonic magnetization response is described by Equation 8.

(8) $$u^{P}=G(x)\cdot f(x)=\frac{2A}{\pi }\cdot \frac{\omega }{4x^{2}+\omega^{2}}\frac{\pi }{30}{\left(\frac{m_{0}\mu_{0}}{k_{B}T}\right)}^{3}A_{H}A_{L}^{2}\times \left\{(\;f_{H}-2f_{L})\cos[2\pi (\;f_{H}+2f_{L})t]+\frac{3}{2}\pi \right\}$$

**Figure 5:**
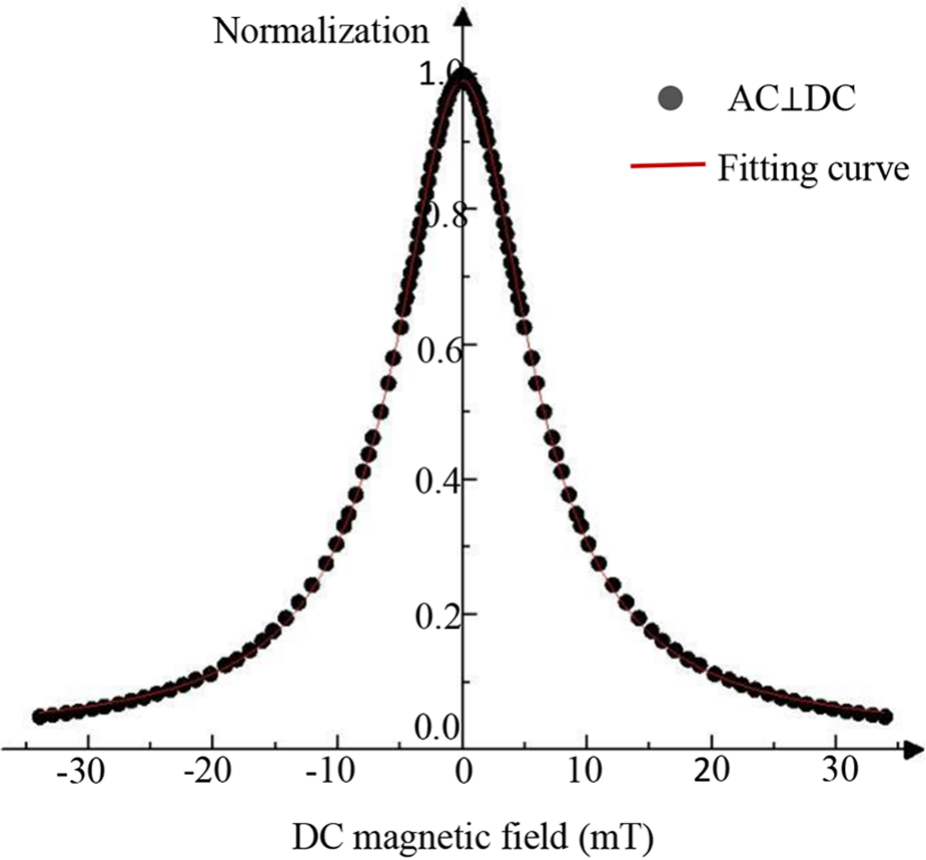
DC magnetic field data normalization process (normalized number: 117).

As illustrated in Figure 5, the normalized experimental ***G*(*x*)** function exhibited strong overlap with the harmonic response curve under ***B_ac_***⊥***B_dc_*** conditions, yielding a linear correlation coefficient (***R*^2^**) of 0.99. This high correlation validates the applicability of the measured *G*(*x*) function to the third-harmonic signal behavior within our narrowband MPI system under ***B_ac_***⊥***B_dc_*** conditions. Consequently, this configuration was adopted for subsequent experiments.

## 3 System development

To validate the feasibility of mixed-frequency harmonic magnetization response-based magnetic particle imaging, a dedicated MPI platform was developed. This platform served as a testbed for evaluating the proposed technique and its potential for future applications.

### 3.1 Gradient coil

To localize magnetic nanoparticle detection within the imaging region, a specific magnetic field gradient is applied to create field-free points (FFPs) or field-free lines (FFLs). These points serve as focal points for magnetic nanoparticles, which exhibit a pronounced response to the driving field. By saturating the magnetization of nanoparticles outside the focal field region, image clarity and accuracy are enhanced. This principle underlies the narrowband MPI system design presented in this study.

The gradient coil configuration features a perpendicular arrangement of AC excitation and DC gradient magnetic fields. Four gradient coils are symmetrically positioned at the four corners of the imaging space, with the system's center serving as the reference point. The centers of each coil are equidistant from one another, and the spatial coordinate system is established with the system's center as the origin. The gradient coils are located at the corresponding corners of the positive and negative directions of the *X*, *Y*, and *Z* axes. Two diagonally positioned coils are connected to the same current direction, while the other two diagonally positioned coils are connected to the opposite current direction.

**Figure 6:**
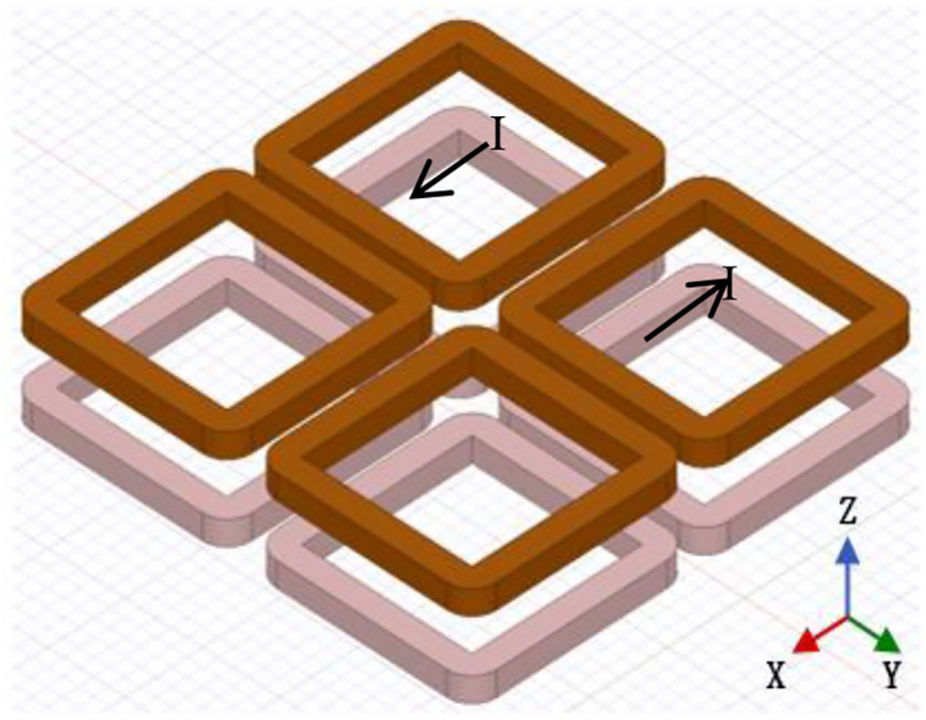
Schematic diagram of the gradient coil.

The gradient coils were wound on four coil formers with a diameter of 40 mm using a single strand of copper wire with a diameter of 1 mm and 318 turns. The average DC resistance of the four gradient coils was measured to be 2.03 Ω. When a current of 4.7 A is passed through the gradient coils, a magnetic field gradient of 0.28 T/m is generated. Figure 6 illustrates the specific arrangement of the gradient coils.

**Figure 7:**
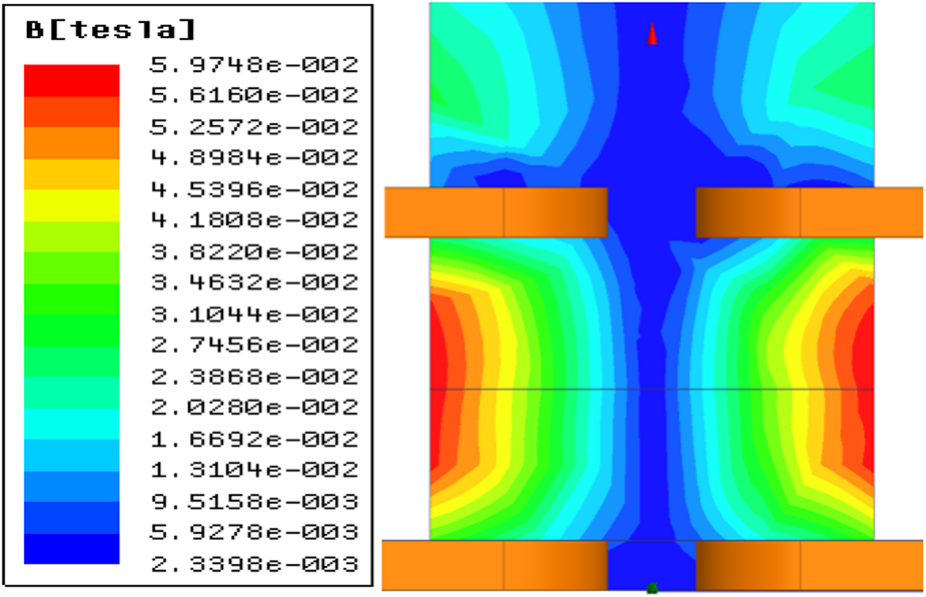
Field free line simulation diagram.

To generate field-free lines (FFLs) along the *z*-axis within a three-dimensional space, an eight-coil system with a planar configuration was developed. A DC current was applied to these coils as depicted in Figure 6. Opposing coil configurations produced gradient magnetic fields of equal magnitude but opposite direction along the *x*, *y*, and *z* axes, intersecting at the coil array's center to form the desired FFL. As distance from the center increases, the *z*-direction magnetic field components from adjacent coils cancel, while substantial *x* and *y* direction gradients emerge. Simulated magnetic field distributions in the *x*-*z* plane (Figure 7) validate this design.

### 3.2 MPI imaging system

**Figure 8:**
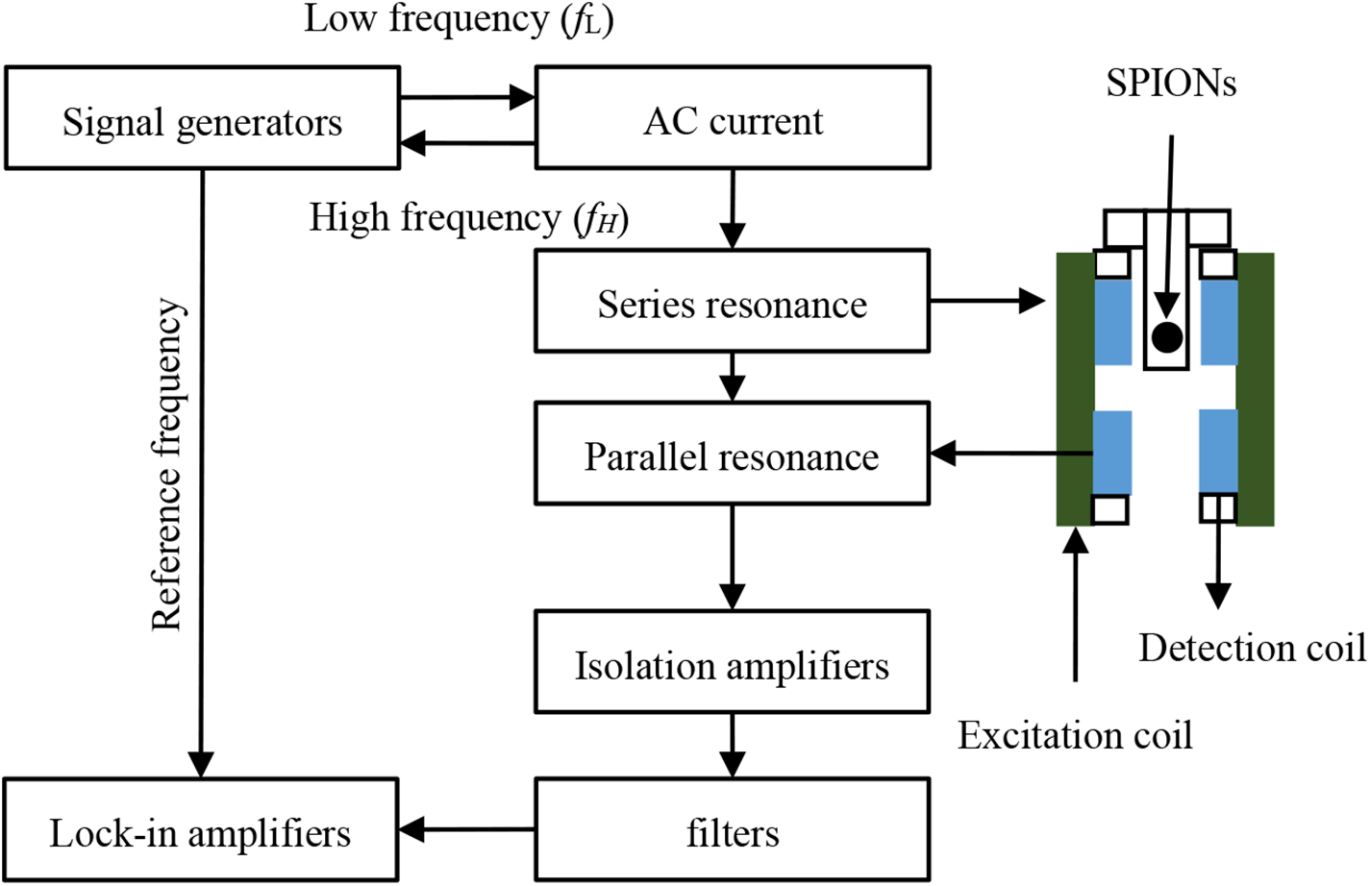
Schematic diagram of a mixed-frequency MPI system.

A mixed-frequency MPI system was constructed, incorporating a gradient coil, excitation coil, analog signal receiver, and peripheral power modules. A mixed-frequency AC magnetic field was generated by applying a mixed-frequency sinusoidal signal to the excitation coil via a resonant circuit. The resulting magnetization of superparamagnetic iron oxide nanoparticles induced a voltage signal captured by a differential detection coil. Subsequent signal processing involved shunt resonant circuit filtering, isolation amplification, and lock-in amplification. The differential detection coil, comprising two identically wound coils connected in reverse series, minimized excitation field coupling and enhanced signal-to-noise ratio. A lock-in amplifier extracted the mixed-frequency harmonic voltage signal, using the signal generator output as a reference. This system architecture is illustrated in Figure 8.

**Figure 9:**
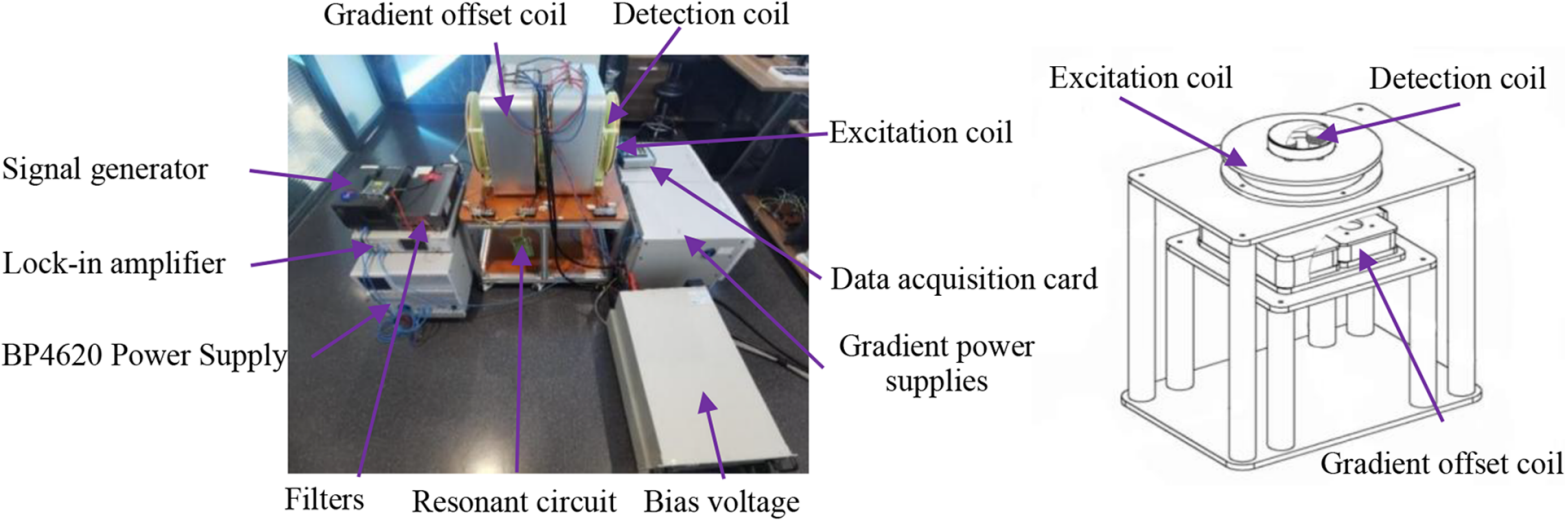
Mixed MPI system structure and coil 3D structure model.

**Figure 10:**
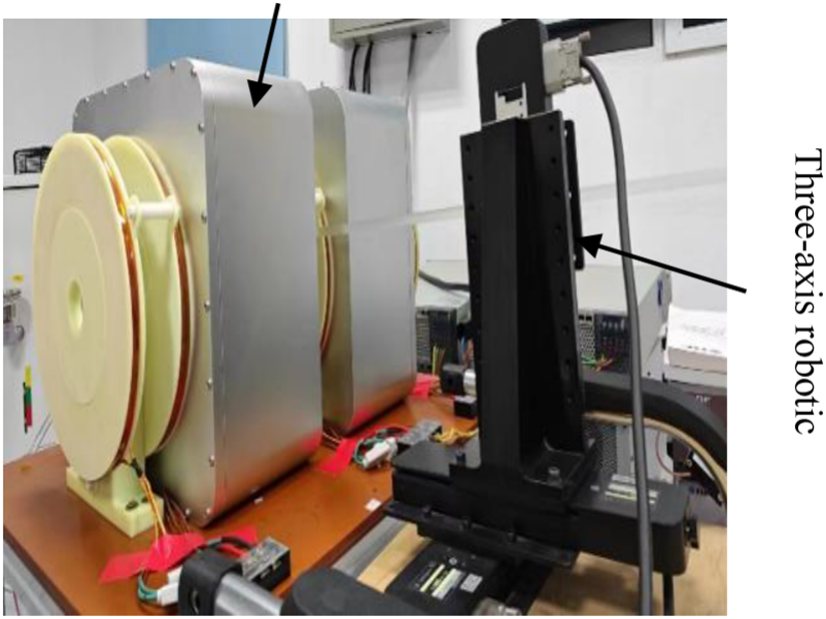
Schematic diagram of MPI mechanical scanning.

The mixed-frequency MPI hardware system comprises a gradient coil, excitation coil, analog signal receiver, and ancillary power modules. To achieve the critical ***B_ac_***⊥***B_dc_*** configuration, the excitation and gradient coils are arranged in a parallel layout. This configuration optimizes system performance and image quality by leveraging the experimental data. The spatial arrangement of system components and the mechanical scanning mechanism are illustrated in Figures 9, 10, respectively.

The system comprises two gradient coil sets (I and II) configured to establish an open field-free line scanning region. A robotic arm facilitates sample manipulation within this area. Excitation and detection coils, strategically positioned to align with the field-free line region, ensure optimal imaging conditions. This open architecture accommodates *in vivo* imaging and continuous monitoring. The detection coil's signal is solely influenced by the gradient field, enabling the use of a strong DC gradient for enhanced spatial resolution while maintaining a low excitation field. To further improve signal-to-noise ratio and image clarity, the system incorporates a cooled medium and harmonic imaging acquisition.

### 3.3 Mechanical scanning

**Figure 11:**
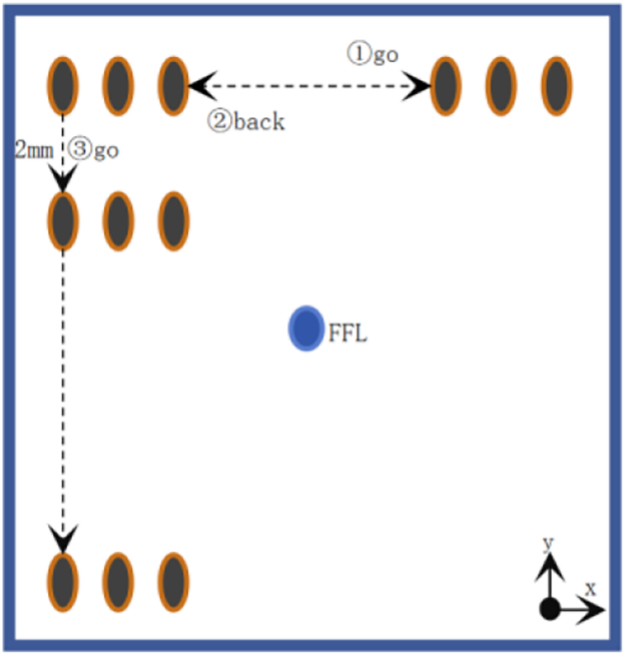
Sample movement trajectory.

To acquire harmonic signals from superparamagnetic nanoparticles, a mechanical scanning approach was employed, wherein the sample was translated through a stationary coil system (comprising excitation, pickup, and gradient coils). The sample trajectory is depicted in Figure 11.

This experiment employed a three-axis robotic arm. Its flexible movement facilitated efficient and adaptable signal field image acquisition. The arm's precise motion control enabled precise control of the sample's scanning path, enhancing data accuracy and experimental repeatability. To precisely manipulate the Resovist sample on the robotic arm, the experiment was controlled using a LabVIEW program. This powerful programming and data acquisition tool allowed real-time monitoring and adjustment of the robotic arm's motion trajectory, ensuring accurate execution of the signal field image acquisition process.

## 4 Imaging system optimization

### 4.1 Enhancement of detection signal

In Magnetic Particle Imaging (MPI) systems, MPI signals are acquired by detecting the nonlinear magnetization response of superparamagnetic materials. To enhance signal detection voltage under the same detection current, this study employs a narrowband detection method that utilizes parallel resonance to improve impedance within a specific frequency band. The resulting detection sensitivity is Equation 9 as follows:

(9) $$\frac{V_{S}}{B_{S}}=QNS2\pi f_{N}$$

Where *S* represents the cross-sectional area of the detection coil, *N* represents the number of turns of the coil, *f_N_* refers to the detection frequency, *B_S_* is the magnetic field strength, and *Q* represents the quality factor of our narrowband resonant circuit. The quality factor can be calculated using the following Equation 10:

(10) $$Q=\frac{\omega L}{R}$$

Where the capacitance in the resonant circuit is determined by the following relation, as follow Equation 11:

(11) $$C=\frac{1}{\omega^{2}L}$$

These equations indicate that higher detection sensitivity can be achieved with a relatively high frequency band and a low equivalent DC resistance (*R*) of the circuit. Furthermore, Enpuku's research (27) demonstrates that cooling the coil to reduce its resistance (*R*) significantly enhances the quality factor (*Q*), resulting in improved system sensitivity.

### 4.2 Noise suppression

While enhancing the system's signal detection capability, an inevitable consequence is an increase in noise within the same frequency band. Although resonant systems effectively mitigate noise interference in non-target frequency bands, noise within the target frequency band remains a challenge. System noise can be broadly categorized into internal and external sources. External noise primarily originates from excitation noise, signal acquisition noise, and environmental noise. Internal noise, on the other hand, is predominantly thermal noise generated by the coil, encompassing equivalent current noise, equivalent voltage noise, and equivalent resistance noise. By effectively reducing these internal and external noise sources, we can significantly improve the sensitivity of the detection coil.

In this experimental system, system noise (*V*_noise_) can be expressed by the following formula Equation 12:

(12) $$V_{\mathrm{noise}}=\sqrt{V_{\mathrm{noise}\text{-}\mathrm{In}}^{2}+V_{\mathrm{noise}\text{-}\mathrm{out}}^{2}}$$

In the preceding equation, *V*_noise-in_ denotes internal system noise, while *V*_noise-out_ represents external system noise. Internal system noise can be further categorized Equation 13 as follows:

(13) $$V_{\mathrm{noise}\text{-}\mathrm{In}}=\sqrt{{\sqrt{S_{I}}}^{2}+{\sqrt{S_{V}}}^{2}+{\sqrt{S_{R}}}^{2}}$$

In the formula above, $\sqrt{S_{I}}$ represents the equivalent current noise, $\sqrt{S_{V}}$ represents the equivalent voltage noise, and $\sqrt{S_{V}}$ represents the equivalent resistance noise of the detection circuit amplifier. Previous research by Takafumi Morishige (28) and others successfully employed a low-noise amplifier to measure $\sqrt{S_{V}}$ and $\sqrt{S_{I}}$. This study followed the same methodology, utilizing a low-noise amplifier to measure $\sqrt{S_{V}}=2\;\mathrm{nV}/\sqrt{\mathrm{Hz}}$ and $\sqrt{S_{I}}=0.15\;\mathrm{pA}/\sqrt{\mathrm{Hz}}$ in the experiment.

Experiments conducted at room temperature revealed that the noise signal generated by the system equivalent resistance noise ($\sqrt{S_{R}}$, which can be considered as thermal disturbances in the detection coil), significantly exceeded $\sqrt{S_{I}}$ and $\sqrt{S_{V}}$. This finding indicates that $\sqrt{S_{R}}$ remains the primary source of internal noise and can be expressed by the following Equation 14 formula (27):

(14) $$\sqrt{S_{R}}=\frac{\sqrt{4k_{B}TR}}{\pi /4D^{2}2\pi fN}$$

In the preceding equation, *k_B_* denotes the Boltzmann constant, *f* represents the signal frequency, *N* signifies the number of coil turns, *D* designates the average diameter of a single coil strand, and *T* is the room temperature (300 K). Substituting these values into the formula at a frequency of f=20.7kHz yields $\sqrt{S_{R}}=17\;\mathrm{nV}/\sqrt{\mathrm{Hz}}$.

As demonstrated above, $\sqrt{S_{R}}$ is the primary source of thermal noise within the coil. To mitigate this thermal noise, we implemented a cooling shell filled with liquid nitrogen surrounding the detection coil in this experiment. This cooling mechanism effectively reduces the temperature of the detection coil, thereby decreasing its resistance and minimizing thermal noise.

Regarding external noise (*V*_noise-out_), it can be further expressed Equation 15 as follow:

(15) $$V_{\mathrm{noise}\text{-}\mathrm{out}}=\sqrt{{\sqrt{S_{E}}}^{2}+{\sqrt{S_{S}}}^{2}+{\sqrt{S_{W}}}^{2}}$$

In the preceding equation, $\sqrt{S_{E}}$ represents the excitation noise, $\sqrt{S_{S}}\;$ denotes the signal acquisition noise, and $\sqrt{S_{W}}$ refers to the external ambient noise. Through techniques such as circuit processing and physical shielding, these noise sources can be significantly minimized and are therefore considered negligible for this study.

### 4.3 Spatial resolution improvement based on mixing excitation

**Figure 12:**
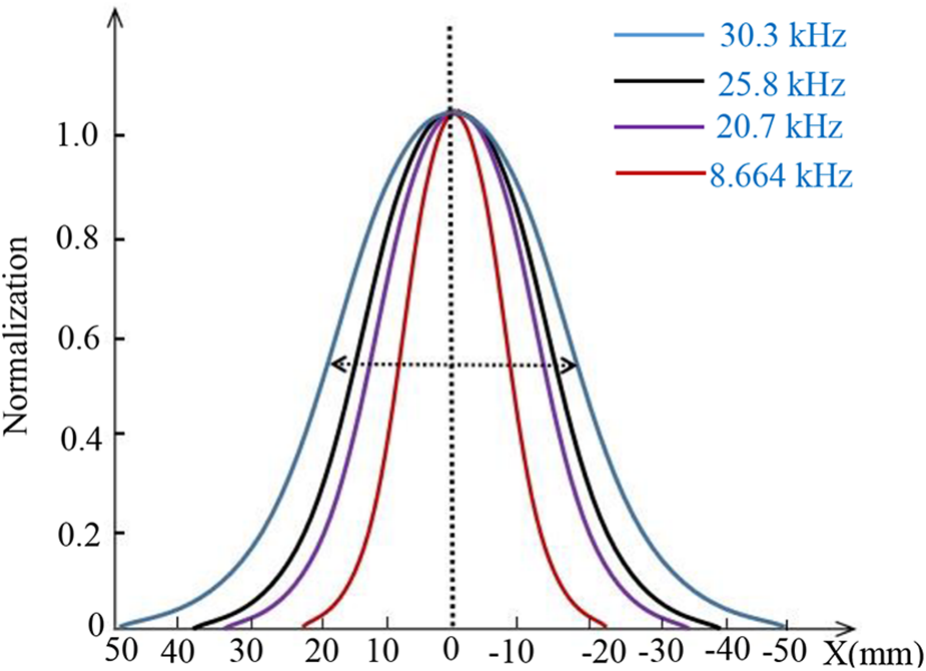
Normalized peaks of each waveform of the three-harmonic signal for different excitation fields in the same gradient field.

A correlation exists between spatial resolution and excitation field strength at a constant gradient field strength. To investigate this relationship, experiments were conducted. Figure 12 displays the waveform of the third harmonic signal generated by an MNP sample containing 100 µg as the FFP (or MNP sample) is moved along the *x*-direction at *y* = 0. The results are presented for various excitation fields while maintaining a constant gradient. In the figure, each waveform peak of the third harmonic signal is normalized. These results demonstrate that MPI imaging exhibits improved spatial resolution when the excitation field strength is relatively low. On the other hand, when the excitation field strength is lower, the signal distribution more closely resembles the original sample size.

**Figure 13:**
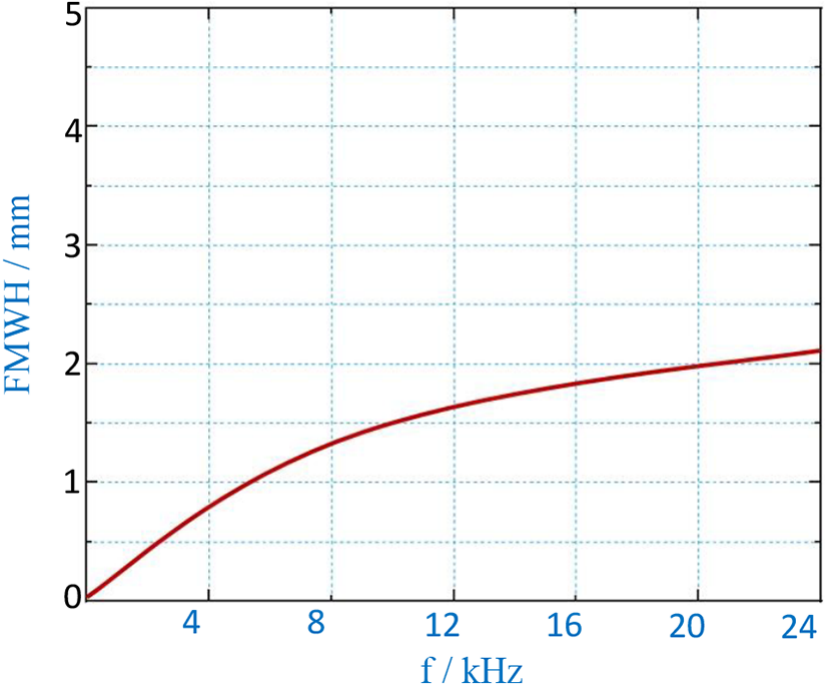
Measured FWHM vs. excitation field.

Figure 13 illustrates the relationship between the measured Full Width at Half Maximum (FWHM) and varying excitation field strengths at a constant gradient field strength. As the excitation field strength increases, the FWHM of MPI imaging exhibits a rapid increase. Conversely, when the excitation field strength is lower, the decrease in FWHM is less pronounced. With a gradient field strength of *G* = 0.12 T/m and an excitation frequency of *f* = 8.664 kHz, a FWHM of approximately 1.5 mm can be achieved.

## 5 Magnetic nanoparticle imaging experiments

### 5.1 Superparamagnetic iron oxide nanoparticle

Tracer selection in magnetic resonance imaging (MRI) is crucial for achieving optimal performance and ensuring safety. An ideal tracer should possess a combination of desirable characteristics: high magnetic susceptibility, a narrow size distribution, and biocompatibility. To generate a robust signal, the tracer must exhibit high magnetization and rapid magnetic relaxation. Furthermore, a narrow particle size distribution is essential for accurate imaging and quantification. Biocompatibility, often achieved through encapsulation in materials like polyethylene glycol (PEG), is critical for *in vivo* applications.

Resovist, a commercially available magnetic nanoparticle, was chosen for this study due to its favorable performance and safety profile for human use. Resovist consists of dextran-coated clusters of iron oxides, exhibiting suitable magnetic properties. This superparamagnetic iron oxide nanoparticle (SPION) sample, a commercial MRI T2 contrast agent, has a hydrodynamic diameter of approximately 60 nm, an average internal multinuclear particle size of approximately 21 nm, and a mononuclear diameter of 3–5 nm. The Resovist sample was obtained from Warmer Ltd. under the kit product number 22P01. Resovist has received approval for clinical use from both the European Medicines Agency and the Japanese Medicines and Medical Devices Agency.

### 5.2 Mechanical scanning imaging results of MNPs

**Figure 14:**
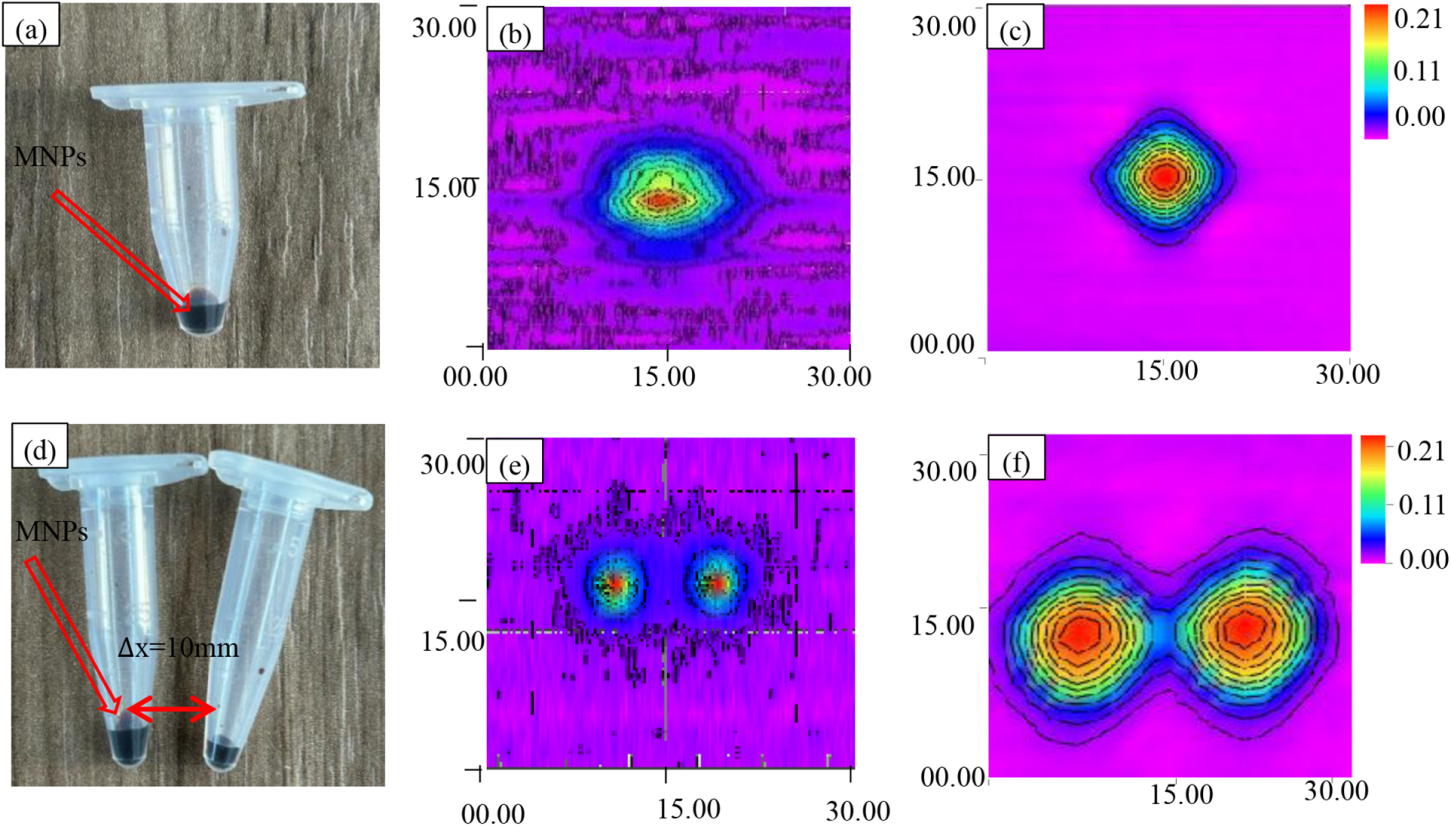
Scanning imaging of MNPs. **(a)** Magnetic nanoparticle samples from a single point source. **(b)** Single-point voltage contour before improvement. **(c)** Single-point voltage contour after improvement. **(d)** Dual point source magnetic nanoparticle sample. **(e)** Dual point voltage contour before improvement. **(f)** Dual point voltage contour after improvement.

To assess system performance, imaging comparisons were conducted using single- and double-point magnetic nanoparticle (MNP) samples (Figure 14a,d). A single-frequency excitation system operating at 20.7 kHz was employed for imaging. A 100 μg MNP sample was positioned within a conical single-element device (diameter ≈ 6 mm, height ≈ 30 mm) at *z* = 0, 15 mm below the detection coil. During the experiment, 2 A current was applied to the gradient coil, generating a magnetic field gradient of 0.12 T/m.

Following the same protocol, two identical 100 μg MNPs were positioned 15 mm below the pickup coil at *z* = 0, *y* = −5 and *z* = 0, *y* = 5, separated by 10 mm for imaging experiments. These configurations served as reference images, depicted in Figure 14b,e.

Subsequently, a mixed-frequency (*f_H_* + 2*f_L_* = 8.664 kHz) harmonic magnetic particle imaging (MPI) system was employed, and the results are shown in Figure 14c,f. For both MPI imaging methods, the third harmonic component was utilized as the harmonic signal for image reconstruction.

To comprehensively assess imaging characteristics, the point spread function (PSF) was employed. Serving as a benchmark, the PSF quantifies the relationship between sample concentration and image resolution, providing essential data for image reconstruction. Rigorous PSF analysis facilitated the construction of the system matrix, a critical component of the reconstruction algorithm. To ensure PSF accuracy, a high-concentration MNP sample was precisely positioned at the gradient field center, and multiple imaging experiments were averaged. The resulting PSF, visualized in Figure 14, serves as a foundation for subsequent image reconstruction and analysis.

Figure 14b presents conventional single-frequency magnetic particle imaging (MPI) results for a 30 mm × 30 mm field of view. While the image reveals the general morphology of the single-point sample, it suffers from a low signal-to-noise ratio, resulting in a spatial resolution of 2 mm and limited image clarity.

In contrast, Figure 14e demonstrates the enhanced capabilities of the proposed mixed-frequency harmonic MPI system. This system utilizes a 1 mT amplitude excitation field combined with 8.460 and 0.102 kHz frequencies, significantly reducing noise to approximately 1.5 μV and improving spatial resolution to 1.5 mm. The resulting image in Figure 14e clearly depicts the single-point sample with improved detail, highlighting the system's ability to enhance image quality and resolution.

### 5.3 Dual sample imaging results

**Figure 15:**
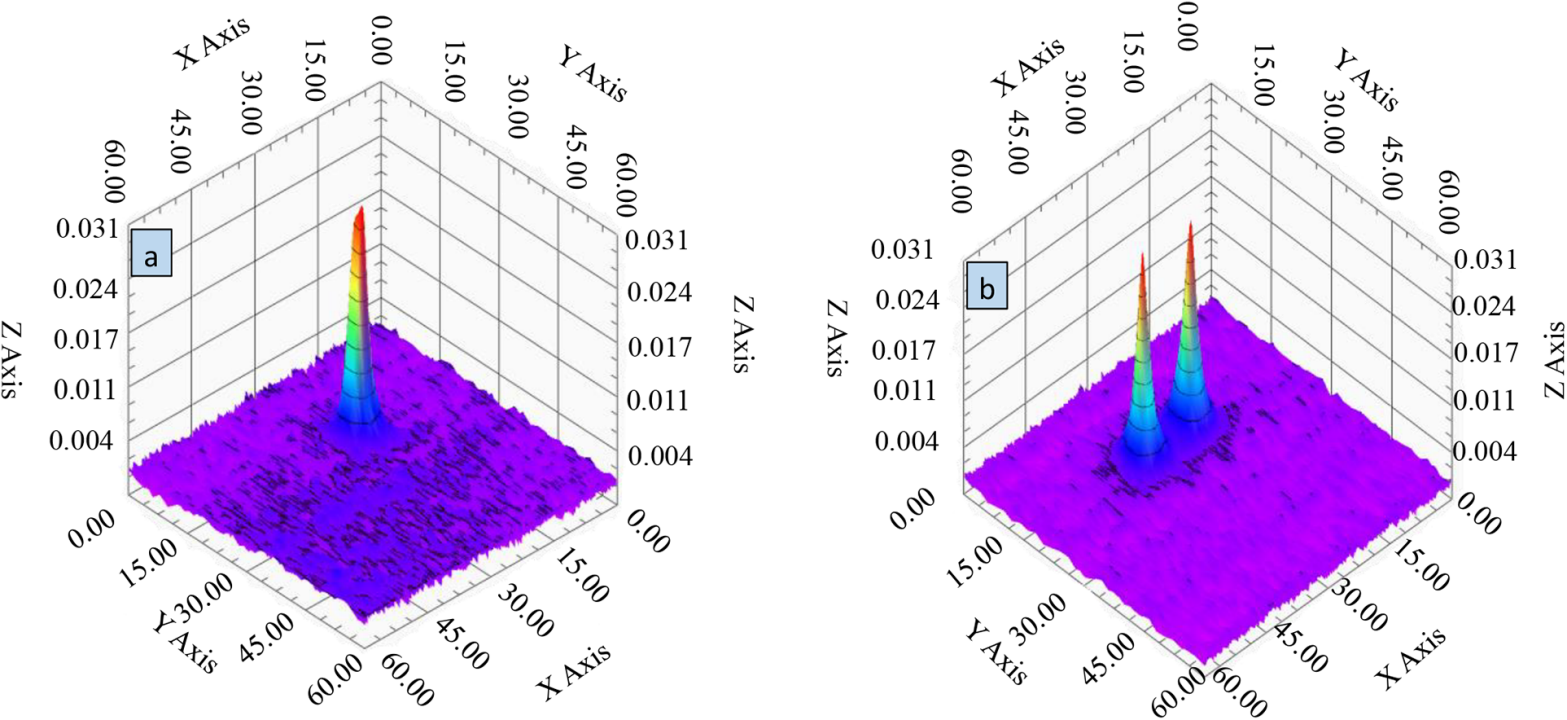
3D voltage cloud of MNP particle samples. **(a)** Single sample 3D voltage cloud. **(b)** Two-sample three-dimensional voltage cloud.

To assess the spatial imaging capabilities of the mixed-frequency harmonic response narrowband MPI system, imaging experiments were conducted using the sample configurations depicted in Figure 14a,d. The resulting three-dimensional voltage cloud is presented in Figure 15.

Figure 15 demonstrates the system's ability to differentiate between two spatially separated samples, with distinct voltage peaks evident in the 3D visualization. This result confirms the narrowband MPI system's high spatial resolution and its capacity to accurately discern closely positioned samples of the same type. The voltage cloud in Figure 15 also reveals the spatial distribution of the voltage response, which correlates directly with gradient field variations. Notably, the clear spatial separation of the dual samples and their unique distribution patterns within the imaging region underscore the system's potential for high-density sample imaging and analysis. These findings underscore the system's ability to distinguish neighboring samples and resolve fine structures, demonstrating its potential for advanced clinical imaging applications.

## 6 Discussion

To evaluate the impact of mixed-frequency harmonic excitation on imaging performance, comparative imaging experiments were conducted using single-point and dual-point MNPs (Figure 14a,d). Conventional single-frequency MPI imaging results (Figure 14b,e) served as a baseline for comparison with the proposed mixed-frequency harmonic MPI system (Figure 14c,f). These results demonstrate the improved image quality and resolution achieved with the mixed-frequency approach.

To further assess spatial resolution, the point spread function (PSF) was employed. The PSF quantifies the relationship between sample concentration and image resolution, providing essential data for image reconstruction. By analyzing the PSF dataset, the system matrix, crucial for image reconstruction, was constructed. To ensure PSF accuracy, a high-concentration MNP sample was precisely positioned and imaged multiple times. The resulting PSF, visualized in Figure 15, demonstrates the system's ability to differentiate between neighboring samples and resolve fine structures.

## 7 Conclusion

This study presents the design of a MPI signal acquisition system that utilizes a mixed-frequency harmonic nonlinear magnetization response. This design aims to improve the signal-to-noise ratio (SNR) and achieve highly sensitive detection of superparamagnetic nanoparticles (SPIONs) while minimizing power consumption. The following key findings are presented:

(1) The system employs a mixed-frequency magnetization approach to excite and record the SPION response. This involves a constructed open-ended narrowband signal detection architecture that utilizes a high-amplitude low-frequency magnetic field superimposed on a low-amplitude high-frequency field. This unique approach facilitates the excitation of a strong nonlinear magnetization response in SPIONs within a low-power AC magnetic field environment.
(2) The proposed open narrowband signal detection architecture enhances the mixed-frequency harmonic signals generated by the nanoparticles. Simultaneously, the narrowband detection technique significantly improves SNR by effectively suppressing fundamental and ambient noise. Experimental results demonstrate noise reduction to approximately 1.5 µV across a 30 mm × 30 mm imaging area. Furthermore, with a 3 mm spacing between samples, the imaging experiments achieve an unreconstructed spatial resolution of 1.5 mm.
(3) The open narrowband mixed-frequency harmonic magnetization MPI technique effectively achieves high-sensitivity signal acquisition with high spatial resolution. Experimental results validate the feasibility of this technical scheme, providing novel design ideas and technical pathways for future MPI systems.

## Data Availability

The original contributions presented in the study are included in the article/Supplementary Material, further inquiries can be directed to the corresponding authors.
